# Dose Correlation of *Panax ginseng* and *Atractylodes macrocephala* Koidz. Drug Pairs in the Chinese Medicine Prescription Based on the Copula Function

**DOI:** 10.1155/2021/9933254

**Published:** 2021-08-24

**Authors:** Wei Lin, Mingyue Zheng, Yunhui Chen, Qian He, Adeel Khoja, Mingyue Long, Jiaxin Fan, Yiwen Hao, Chaomei Fu, Peng Hu, Ke Wang, Jianhua Jiang, Xuan Zhao

**Affiliations:** ^1^School of Management, Chengdu University of Traditional Chinese Medicine, Chengdu 611137, China; ^2^Adelaide Medical School, University of Adelaide, Adelaide 5005, Australia; ^3^School of Health and Rehabilitation, Chengdu University of Traditional Chinese Medicine, Chengdu 611137, China; ^4^College of Clinical Medicine, Chengdu University of Traditional Chinese Medicine, Chengdu 611137, China; ^5^No. 1 Orthopedics Hospital of Chengdu, Qingyang District Medical Center, Chengdu 610000, China; ^6^School of Pharmacy, Chengdu University of Traditional Chinese Medicine, Chengdu 611137, China; ^7^School of Big Data and Artificial Intelligence, Chengdu Technological University, Chengdu 611730, China

## Abstract

**Objective:**

*Panax ginseng* and *Atractylodes macrocephala* Koidz. (AMK) are widely used in treating various diseases; however, research is insufficient on measuring the relationship that exists by combining this drug pair using the copula function.

**Methods:**

In this study, 279 traditional Chinese medicine prescriptions containing the *Panax ginseng* and AMK drug pair were extracted from the prescription database for three types of screened indications, namely, diabetes mellitus, diarrhea, and insomnia. Following the principle of dose conversion, each dynasty unit was uniformly converted into a modern unit. Then, the kernel density distribution of *Panax ginseng* and AMK was fitted with their empirical distribution functions. Finally, the optimal copula function was selected from the copula function family as a *t*-copula function.

**Results:**

The empirical distribution and probability density functions of *Panax ginseng* and AMK were obtained. From the results, their Kendall rank correlation coefficients with indications of diabetes mellitus, insomnia, and diarrhea were 0.8689, 0.7858, and 0.7403, whereas their Spearman rank correlation coefficients were 0.9563, 0.9276, and 0.8958. Results also indicated that the use of the *t*-copula function can better reflect the correlation between *Panax ginseng* and AMK doses.

**Conclusion:**

From the three indications, the dose between *Panax ginseng* and AMK was positively correlated. This study, therefore, confirms the medicinal principle of Chinese medicine “combining” from the perspective of mathematical statistics. Results from this study are practical to evaluate the correlation between the drug pair doses, *Panax ginseng* and AMK, using the copula function model, which provides a new model for the scientific explanation of compatibility for Chinese medicines. This study also provides a methodological basis for more drug measurement studies and clinical medications.

## 1. Introduction

*Panax ginseng* (*Panax ginseng* C. A. Mey.) is a perennial herb of Panax ginseng in the Araliaceae family of Umbelliferae [1]. Its medicinal functions include strengthening the spleen and lungs, as well as relieving adversity. It is an essential medicine for invigorating fatigue and is first recorded in the *Compendium of Materia Medica* [2]. Modern pharmaceutical research has proved that Rb1, Rb2, and Rb3 in *Panax ginseng*'s saponin fraction can lower blood sugar, and its effects are similar to those of an insulin sensitizer [3–5]. Alternatively, *Atractylodes macrocephala* Koidz. (AMK) is a perennial herb of the genus *Atractylodes* in the Compositae family, which has functions of strengthening the spleen and replenishing qi, drying dampness and diuresis, antiperspirants, and antifetuses. Studies have also found that AMK polysaccharides can effectively reduce fasting blood glucose in db/db2 diabetes mellitus mice by increasing insulin sensitivity as well, thereby improving glucose tolerance [6].

Recently, researchers have used emerging technologies to explore the combination of *Panax ginseng* and AMK in traditional Chinese medicine (TCM). Studies have shown that *Panax ginseng* and AMK can be used to treat diabetes mellitus, stool slippage, insomnia, etc. [7]. Furthermore, the *Jingfang Sijunzi* decoction (comprising *Panax ginseng*, AMK, *Poria*, and licorice) has been proven effective in treating diabetes [8]. This decoction mainly plays a role in treating diabetes mellitus by participating in apoptosis, oxidative stress, inflammation, endothelial cell proliferation, etc. [9]. Additionally, *Panax ginseng* and AMK are commonly used combination drugs proven to lower blood glucose [10]. Similarly, *Shenling Baizhu San* (white lentils, AMK, *Platycodon*, lotus seeds, and *Panax ginseng*) can significantly improve the fasting and 2 h postprandial blood glucose levels of patients with diabetes mellitus and diarrhea [11]. Also, according to *Jingyue Quanshu*, the Shenshu decoction (*Ginseng*, *Atractylodes*, *Astragalus*, etc.) is used for treating symptoms, such as diarrhea, vomiting, and tremors [12]. Combining *Panax ginseng* and AMK can also be used to treat insomnia by regulating the intestinal flora [11].

In the compatibility of TCM research, dose plays a crucial role in efficacy, and the drug compatibility of different doses produces widely different results. Thus, current statistical methods for evaluating the compatibility of TCM mainly include data mining, cluster analysis, factor analysis, principal component analysis, machine learning, and neural network-based method [13–16]. Text data mining (or text mining) has also been made among the most active research subfields in data mining [17]. Among these valuable text data mining approaches, the copula function has been shown to be outstanding. The proposal of copula function-related theories can be traced back to 1959, where Sklar used the theory to connect multivariate distributions with the copula function [18]. It is a type of function that connects joint distribution functions with their respective marginal distribution functions, which can then be used to determine the dependence between random variables on a nonparametric measurement. Furthermore, the copula function is practical for analyzing the correlation between variables when it cannot determine whether the traditional linear correlation can correctly measure the correlation between variables.

As an effective data analysis method, the copula function has also been used to study the dose relationship between clinical combinations of Western medicines [19], evaluate the clinical relevance of combination therapy [20], and compare the effects of two diagnostic methods [21]. However, which copula function should be used is determined after measuring the effect. Thus, the copula regression approach was used to model toxicity attributes and estimate the maximum tolerance dose curves on the basis of model parameters, thereby searching for drug doses on the basis of the current dose of another drug [22]. A research by Mónica and Stephen identified the combined response of EQ-5D-3L and EQ-5D-5L in patients with rheumatic diseases in combination with the copula function of normally mixed edges [23]. Similarly, Houede et al. adopted the Gaussian copula approach to obtain a combined distribution for selecting the best dose pair for the combined application in chemotherapy drugs and biological agents [24]. Additionally, the copula method is more sensitive and accurate than traditional statistical methods because it can accurately capture the time waves that depend on the structure and simultaneously visualize the dependence levels of these waves within a specific signal [25].

The drug pair (*Panax ginseng*-AMK) has been widely used in the past prescriptions of doctors, and the dose in these different prescriptions is yet to be clearly stipulated. Also, most of the current studies on drug pairs compare the chemical composition of these drugs, including their absorption, metabolism, or changes in their pharmacological effects. However, the lack of research on the compatibility of drug pairs with dose makes it difficult to show a correlation between drug pairs in metering applications. In a previous study, we innovatively used the copula function to study the correlation between Danggui (*Angelica sinensis*) and Chuanxiong (*Ligusticum*) to find a positive correlation between these two drugs [26]. The new method proved to be one of the best choices for exploring the quantitative relationship between the two drugs on the basis of different indications.

Therefore, this study conducted dose correlation analysis research on *Panax ginseng*-AMK dose from data mining and mathematical statistics. First, we collected prescriptions of TCM containing this pair of prescriptions from self-built prescription libraries, after which we mathematically described the dosages of these two drugs in the three groups of prescriptions as indicated for diabetes, diarrhea, and insomnia. Subsequently, we evaluated the correlation between doses of *Panax ginseng* and AMK present in the medications for treating those three indications, thereby establishing a copula function model analysis for this pair that can be adopted for future studies.

## 2. Materials and Methods

**Figure 1:**
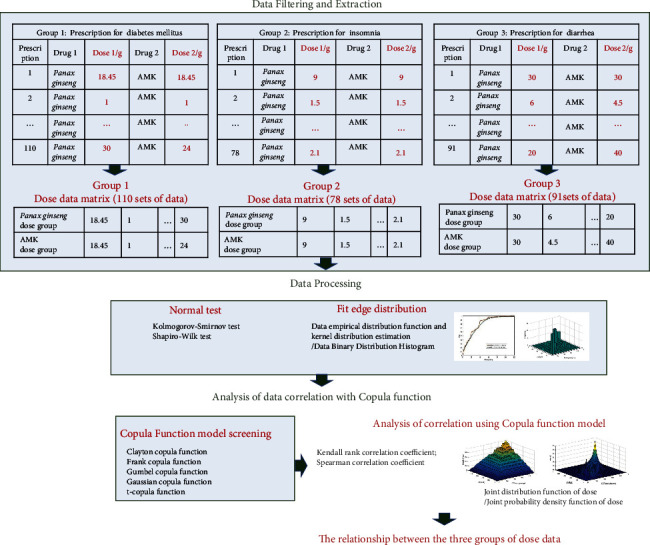
The study process.

The data collection method was based on text mining of existing TCM information sources [17]. Here, we collected major prescription books from the Qin and Han Dynasties and Tang, Song, Ming, and Qing Dynasties to modern times, including classic prescriptions, prescriptions written by some well-known doctors, local prescription for medicines, selected collections of local TCM prescriptions, medical journals, and TCM textbooks. Some prescriptions were also selected from 414 famous doctors in modern times, including some hospitals and pharmaceutical factories. Finally, 51083 prescriptions (the entire database) were included in our prescription database [26].  Figure 1 shows the study process.

### 2.1. Data Extraction

Diabetes is known as “Xiaoke” in TCM, and there were records of *Panax ginseng*-AMK for treating diabetes as early as in “*Neijing*.” In modern medicine, the *Panax ginseng*-AMK drug pair is widely used in treating diabetes, including *Shenling Baizhu* pills, *Ginseng Baizhu San*, and Sijunzi decoction [5]. Consequently, diabetes was chosen in this study. *Panax ginseng*-AMK is the most important drug pair for invigorating the spleen and qi. Furthermore, it is an important drug pair for treating sleepiness of the limbs, poor diet, and diarrhea [9]. Thus, diarrhea was also selected in this study. *Panax ginseng*-AMK for invigorating the spleen and qi is widely used in treating insomnia as well, such as the “Guipi decoction.” So, insomnia was also selected in this study. Furthermore, diarrhea and insomnia are common complications of diabetes [5, 6], and the *Panax ginseng*-AMK prescription has a significant effect on these three indications. According to our data mining results, 110 prescriptions exist for diabetes, 78 for insomnia, and 91 for diarrhea, which met the requirements of the copula function data analysis (Figure 1).

Therefore, according to the following four conditions, we screened prescriptions containing the *Panax ginseng* and AMK pair from the prescription library. The screening conditions are as follows: first, the prescription must contain *Panax ginseng* and AMK; second, different dosage forms can lead to excess dispersion of the drug pair data, so only data presenting the decoction formula were selected [27]; third, we selected prescriptions from the Ming and Qing Dynasties; fourth, only studies using ginseng and AMK as the target drugs for common clinical indications of diabetes, insomnia, and diarrhea, including studies prescribing for treating these three indications, were selected, respectively.

Based on full consideration of representativeness, availability, and accuracy of the dosage indicators of *Panax ginseng* and AMK, cases were selected into the model. Therefore, 110 (diabetes), 78 (insomnia), and 91 (diarrhea) cases, respectively, were selected. Furthermore, because of the inconsistency of these prescription units, the conversion relationship between measurement units of different ages and modern measurement units was different. Also, the conversion of drugs to doses was conducted following the conversion principle [28] (Tables 1 and 2).

After data screening, descriptive statistics were conducted on doses of *Panax ginseng* and AMK from each prescription group. Also, to discuss the relevance of *Panax ginseng* and AMK, we established a prescription database, including diseases, prescription names, prescription details, and prescription sources.

For diabetes mellitus treatment, the median dose (95% CIs) of *Panax ginseng* was most concentrated in 20.0 (18.6–28.2), whereas the median dose of AMK was 20.0 (20.8–29.9). However, for insomnia treatment, the median dose (95% CIs) of *Panax ginseng* was 19.2 (19.6–27.3), whereas the AMK dose was 9.2 (19.2–27.0). Also, for diarrhea treatment, the median dose (95% CIs) of *Panax ginseng* was 15.5 (17.5–25.2), whereas the AMK dose was 30.0 (21.0–29.6) (Table 3). Overall, we found that the compositions of *Panax ginseng* and AMK were similar, but they had different clinical manifestations because of their different dosages.

### 2.2. Normality Test

The Kolmogorov–Smirnov [29] or Shapiro–Wilk tests [30] were used to screen out the drug pair in the prescriptions for diabetes mellitus, insomnia, and diarrhea treatments. We also tested the normality of the doses of *Panax ginseng* and AMK in selected prescriptions for these three indications (Table 4).

The results showed that the *P* values of the Kolmogorov–Smirnov test for different indications were all less than the significance level of 0.01, and the data did not conform to the normal distribution.

### 2.3. Data Fitting Marginal Distribution

**Figure 2:**
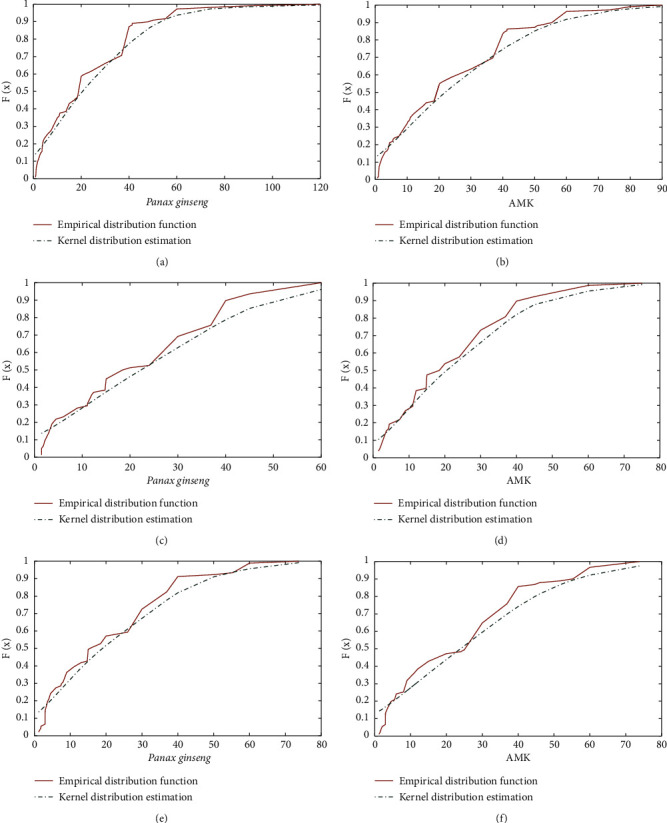
*Panax ginseng* and AMK kernel distribution estimations: (a) *Panax ginseng* (treatment for diabetes mellitus), (b) AMK (treatment for diabetes mellitus), (c) *Panax ginseng* (treatment for insomnia), (d) AMK (treatment for insomnia), (e) *Panax ginseng* (treatment for diarrhea), and (f) AMK (treatment for diarrhea).

**Figure 3:**
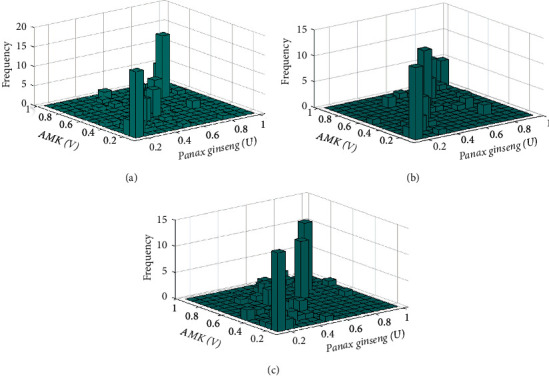
Histograms of the frequency distributions, *Panax ginseng* (*u*) and AMK (*v*): (a) diabetes mellitus, (b) insomnia, and (c) diarrhea.

**Figure 4:**
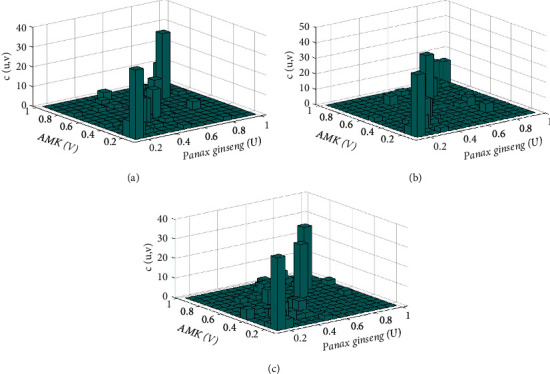
Histograms of the probability distributions, *Panax ginseng* (*u*) and AMK (*v*): (a) diabetes mellitus, (b) insomnia, and (c) diarrhea.

The KS density function was used to calculate the marginal distributions of *Panax ginseng* and AMK, respectively. To avoid errors caused by the continuity and ensure smoothness of the empirical distribution function, the kernel distribution function was also used to fit the empirical distribution function [31]. Results indicated that the doses of *Panax ginseng* and AMK of the three groups of prescriptions had a good fit between the kernel and empirical distribution functions (Figure 2). Also, after determining their respective marginal distributions, the (*U*, *V*) binary distribution histograms of *Panax ginseng* and AMK doses in the three groups of prescriptions were obtained. Figures 3 and 4 show the *Panax ginseng* and AMK doses in the three groups of prescriptions. As shown, the AMK dose had a strong tail correlation. Furthermore, from the frequency distribution histogram, it can be found that the data had asymmetric tail characteristics. Hence, the copula function was used to conduct a comprehensive analysis of the nonlinear relationship between *Panax ginseng* and AMK.

### 2.4. Use of the Copula Function to Analyze Drug Dose Correlations

The copula function is a type of function that connects the joint and the respective marginal distribution functions. It can also be used to determine the dependence between random variables on a nonparametric measurement. Thus, when it is impossible to determine whether the traditional linear correlation coefficient can correctly measure the correlation relationship between variables, the copula function is helpful for analyzing the correlation relationship between these variables. It can also capture the nonlinearity and asymmetry, including the tail correlation between variables.

Furthermore, the copula function is a connection function that connects the joint distribution function *F*(*x*_1_, *x*_2_,…, *x*_*N*_) of the random vector *X*_1_, *X*_2_,…, *X*_*N*_, with its respective marginal distribution functions *F*_*X*1_(*x*_1_), *F*_*X*2_(*x*_2_),…, *F*_*XN*_(*x*_*N*_) [19], that is, *C*(*u*_1_, *u*_2_,…, *u*_*N*_).

(1) $$\begin{array}{c} F\left(x_{1},x_{2},\ldots ,x_{N}\right)=C\left\{F_{X1}\left(x_{1}\right),F_{X2}\left(x_{2}\right),\ldots ,F_{XN}\left(x_{N}\right)\right\}. \end{array}$$

The advantage of the copula function is that it can judge the correlation between variables and is more flexible in choosing correlation measurement methods. For the binary copula function, let *F*(*x*_1_, *x*_2_) be a binary joint distribution function, and its marginal distribution is *F*(*x*_1_), *F*(*x*_2_), and then a copula function *C*(*u*_1_, *u*_2_) is obtained that satisfies

(2) $$\begin{array}{c} F\left(x_{1},x_{2}\right)=C\left(F_{x1},F_{x2}\right). \end{array}$$

Therefore, if *F*(*x*_1_) and *F*(*x*_2_) are continuous functions, then *C*(*u*_1_, *u*_2_) is uniquely determined. Conversely, if *F*(*x*_1_) and *F*(*x*_2_) are univariate distributions, then the copula function is *C*(*u*_1_, *u*_2_). Hence, *F*(*x*_1_, *x*_2_) determines *F*(*x*_1_, *x*_2_) = *C*(*F*_*x*1_, *F*_*x*2_), which is the joint distribution of the marginal distribution, *F*(*x*_1_), *F*(*x*_2_) [21].

This study selected the best copula function from the Archimedes (Gumbel copula function, Clayton copula function, and Frank copula function) and the elliptical copula function families (Gaussian copula function and *t*-copula function) and then described the relationship between *Panax ginseng* and AMK doses. Various copula and empirical distribution functions were also used to solve the squared Euclidean distance using the square Euclidean distance algorithm. Thus, the optimal copula function selected under this standard was the one with the shortest distance between the corresponding empirical copula functions.

### 2.5. Explanation of the Copula Function Family [32]

#### 2.5.1. The Archimedes Copula Function Family

The distribution of the Archimedes copula function is as follows:

(3) $$\begin{array}{c} C\left(u_{1},u_{2},\ldots ,u_{N}\right)=\left\{\begin{array}{cc} \varphi^{-1}\left[\varphi \left(u_{1}\right),\varphi \left(u_{2}\right),\ldots ,\varphi \left(u_{N}\right)\right], & \sum_{i=1}^{N}\varphi \left(u_{i}\right)\leq \varphi \left(0\right),\\ 0, & \text{otherwise}, \end{array}\right. \end{array}$$

where *φ*(*u*_*i*_) was used to generate the Archimedes copula function.

Furthermore, the expression of the binary Gumbel copula function is

(4) $$\begin{array}{c} C\left(u,v\right)=\exp\left({\left(-{\left(-\ln\;u\right)}^{\alpha }+-{\left(-\ln\;v\right)}^{\alpha }\right)}^{1/\alpha }\right), \end{array}$$

where *α* is a parameter, which is more sensitive to the change in an upper tail. Therefore, as *α* gradually increases, if the characteristics of the upper tail changes are more obvious, the correlation between the variables becomes stronger. Hence, the expression for the binary Clayton copula function is

(5) $$\begin{array}{c} C\left(u,v\right)=\max\left({\left({\left(u\right)}^{-\alpha }+{\left(v\right)}^{-\alpha }-1\right)}^{-\left(1/\alpha \right)},0\right), \end{array}$$

where *α* is a parameter that is more sensitive to changes in the lower tail. Therefore, as *α* gradually increases, if the lower tail characteristic changes are more obvious, the correlation between the variables becomes stronger. Thus, the expression for the binary Frank copula function is [33]

(6) $$\begin{array}{c} C\left(u,v\right)=-\frac{1}{\alpha }\ln\left(1+\frac{\left(e^{-\alpha u}-1\right)\left(e^{-\alpha v}-1\right)}{e^{-\alpha }-1}\right), \end{array}$$

where *α* is a parameter, and its tail characteristics have symmetry. So, it cannot capture the asymmetric tail correlation between variables.

#### 2.5.2. Elliptical Copula Function Family [34]

The expression for the binary Gaussian copula function is

(7) $$\begin{array}{c} C\left(u,v,\rho \right)=\int_{-\infty }^{\Phi^{-1}\left(u\right)}\int_{-\infty }^{\Phi^{-1}\left(v\right)}\frac{1}{2\pi \sqrt{1-\rho^{2}}}\exp\left(\frac{-\left(r^{2}+s^{2}-2\rho rs\right)}{2\pi \left(1-\rho^{2}\right)}\right)\text{d}r\text{d}s, \end{array}$$

where Φ^−1^(*u*) and Φ^−1^(*v*) are the inverse functions of the standard normal distribution function of Φ(*i*) and Φ(*v*), *ρ* is the linear correlation coefficient of Φ^−1^(*u*) and Φ^−1^(*v*), and *ρ* ∈ (−1, 1).

Additionally, the expression for the binary *t*-copula function is

(8) $$\begin{array}{c} C\left(u_{1},u_{2};\rho ,v\right)=\int_{-\infty }^{\Gamma_{v}^{-1}\left(u_{1}\right)}\int_{-\infty }^{\Gamma_{v}^{-1}\left(u_{2}\right)}\frac{1}{2\pi \sqrt{1-\rho^{2}}}\left(1+\frac{s^{2}+t^{2}-2\rho st}{v\left(1-\rho^{2}\right)}\right)\text{d}s\text{d}t, \end{array}$$

where Γ_*v*_^−1^(*u*_1_) and Γ_*v*_^−1^(*u*_2_) are the inverse functions of the unary *t*-distribution functions of Γ_*υ*_(*u*_1_) and Γ_*υ*_(*u*_2_), whereas *ρ* is the linear correlation coefficient of Γ_*v*_^−1^(*u*_1_) and  Γ_*v*_^−1^(*u*_2_),  and *ρ* ∈ (−1,1).

#### 2.5.3. Correlation Measure

The correlation coefficient can describe the degree of dependence between two variables and provide a core basis for accurate modeling [34]. Therefore, when measuring the copula function, the Kendall and Spearman rank correlation coefficients are commonly used. Also, when the correlation coefficient (including the Spearman and Kendall rank correlation coefficients) ranges from (−1, 0), a negative correlation exists between the variables. However, when the correlation coefficient is 0, the variables are not correlated [19, 35]. Additionally, when the correlation coefficient range is (0, 1), a positive correlation exists between variables. Hence, the closer the value is to 1, the higher the correlation between the variables is. Thus, when measuring the copula function, Kendall and Spearman rank correlation coefficients are often adopted. The calculation and analysis formula used to calculate the correlation coefficient using the copula function can be found in the published literature [26].

The expression for the Kendall rank correlation coefficient *τ* is

(9) $$\begin{array}{c} \tau =4\int_{0}^{1}\int_{0}^{1}C\left(u,v\right)\text{d}C\left(u,v\right)-1. \end{array}$$

Similarly, the expression of Spearman rank correlation coefficient *r* is

(10) $$\begin{array}{c} r=12\int_{0}^{1}\int_{0}^{1}C\left(u,v\right)\text{d}uv-3. \end{array}$$

If the marginal distribution of random variables *X* and *Y* is *F*(*x*) and *G*(*y*), respectively, the corresponding copula function becomes *C*(*u*, *v*), where *u* = *F*(*x*), *v* = *G*(*y*), and *u*, *v* ∈ [0, 1].

## 3. Results

### 3.1. Dose Relationship between *Panax ginseng* and AMK in the Prescription for Diabetes Mellitus Patients

Among the 110 prescriptions indicated for diabetes mellitus, the *Panax ginseng* and AMK dose groups were substituted into the copula function, after which their corresponding squared Euclidean distances, parameter-estimated values, and correlation coefficients were calculated.

Table 5 reports that the squared Euclidean distance of the *t*-copula function was 0.0090, whereas the parameter estimation *ρ* was 0.9789 and *v* was 6.0473. Thus, the optimal function was the *t*-copula function.

Hence, using the *t*-copula, we can better fit the relationship between *Panax ginseng* and AMK using the following expression:

(11) $$\begin{array}{c} C\left(u_{1},u_{2}\right)=\int_{-\infty }^{\Gamma_{\nu }^{-1}\left(u_{1}\right)}\int_{-\infty }^{\Gamma_{\nu }^{-1}\left(u_{2}\right)}\frac{1}{2\pi \sqrt{1-0.9179^{2}}}\left(1+\frac{s^{2}+t^{2}-1.9578st}{6.0473\left(1-0.9789^{2}\right)}\right)\text{d}s\text{d}t. \end{array}$$

**Figure 5:**
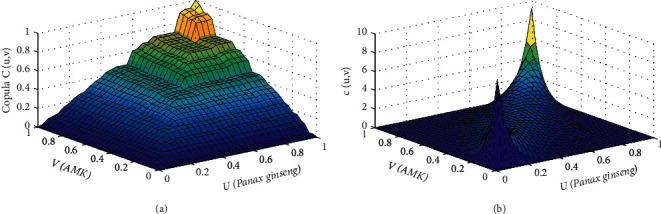
Joint distribution function (a) and probability density function (b) of diabetes mellitus.

The copula *C*(*u*, *v*) is the joint distribution function of *Panax ginseng* and AMK drawn by the Gumbel copula. Also, the marginal distribution function of *Panax ginseng* is *u*(*Panax ginseng*), whereas the marginal distribution function of AMK is *v*(AMK). Furthermore, the tail of the density function in Figure 5 had a thicker morphological feature, which better captured the tail correlation between *Panax ginseng* and AMK. This result also indicated that the model of *Panax ginseng* and AMK established by the *t*-copula function better reflected the correlation between *Panax ginseng* and AMK. Table 5 also shows that the *t*-copula function Kendall rank correlation coefficient was 0.8689, whereas the Spearman correlation coefficient was 0.9563, indicating that *Panax ginseng* and AMK doses had robust nonlinear and positive correlations. Thus, when the doses of *Panax ginseng* and AMK changed significantly, the synergy between the two drugs increased.

### 3.2. Dose Relationship between *Panax ginseng* and AMK in the Prescription for Insomnia Patients

Among the 78 prescriptions with indications of insomnia, doses of *Panax ginseng* and AMK were substituted into the copula function. Next, the corresponding squared Euclidean distances and parameter estimates were calculated (Table 6). Also, since the squared Euclidean distance of the *t*-copula function (Table 6) was at least 0.0108, the optimal function based on this criterion is the *t*-copula function.

However, the estimated parameter *ρ* was 0.9608 and *v* was 4.0089. Therefore, using the *t*-copula better fitted the relationship between *Panax ginseng* and AMK. The expression is as follows:

(12) $$\begin{array}{c} C\left(u_{1},u_{2}\right)=\int_{-\infty }^{\Gamma_{\nu }^{-1}\left(u_{1}\right)}\int_{-\infty }^{\Gamma_{\nu }^{-1}\left(u_{2}\right)}\frac{1}{2\pi \sqrt{1-0.9608^{2}}}\left(1+\frac{s^{2}+t^{2}-1.9360st}{4.0089\left(1-0.9608^{2}\right)}\right)\text{d}s\text{d}t. \end{array}$$

The copula *C*(*u*, *v*) is the joint distribution function of *Panax ginseng* and AMK drawn by the *t*-copula, where the marginal distribution function of *Panax ginseng* is *u*(*Panax ginseng*) and the marginal distribution function of AMK is *v*(AMK). Additionally, the density function tail in the graph had thicker morphological features, which better captured the tail correlation characteristics between *Panax ginseng* and AMK, thereby indicating that the model of *Panax ginseng* and AMK established by the *t*-copula function better reflected the correlation between the drug pairs.

**Figure 6:**
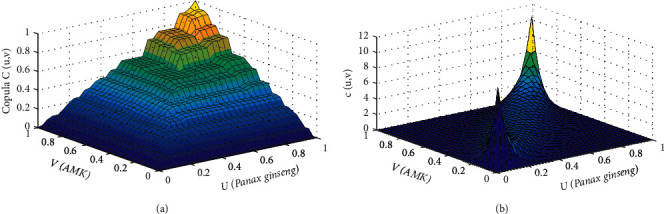
Joint distribution function (a) and probability density function (b) of insomnia.

Table 6 shows that the sum of the Kendall rank correlation coefficients of the *t*-copula function was 0.7858, whereas that of the Spearman correlation coefficient was 0.9276, indicating that *Panax ginseng* and AMK doses had robust nonlinear and positive correlations. Furthermore, when the dose of *Panax ginseng* and AMK changed greatly, the two synergies increased; however, when the dose of only *Panax ginseng* increased, the dose of AMK also increased significantly (Figure 6).

### 3.3. Dose Relationship between *Panax ginseng* and AMK in the Prescription for Diarrhea Patients

Among the 91 prescriptions for diarrhea, the doses of *Panax ginseng* and AMK were substituted into the copula function, after which the corresponding squared Euclidean distances and parameter estimates were calculated (Table 7).

The minimum squared Euclidean distance of the *t*-copula function (Table 7) was 0.0117. Also, the parameter estimates were *ρ* = 0.9179 and *v* = 2.6696. Hence, the optimal function was the *t*-copula function.

Therefore, using the *t*-copula can better fit the relationship between *Panax ginseng* and AMK. The expression is

(13) $$\begin{array}{c} C\left(u_{1},u_{2}\right)=\int_{-\infty }^{\Gamma_{\nu }^{-1}\left(u_{1}\right)}\int_{-\infty }^{\Gamma_{\nu }^{-1}\left(u_{2}\right)}\frac{1}{2\pi \sqrt{1-0.9179^{2}}}\left(1+\frac{s^{2}+t^{2}-1.8358st}{2.6696\left(1-0.9179^{2}\right)}\right)\text{d}s\text{d}t. \end{array}$$

**Figure 7:**
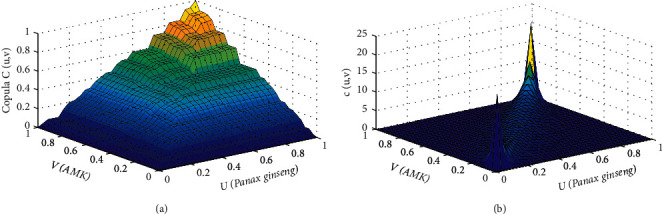
Joint distribution function (a) and probability density function (b) of diarrhea.

The copula *C*(*u*, *v*) is the joint distribution function of *Panax ginseng* and AMK based on the *t*-copula. Therefore, the marginal distribution function of *Panax ginseng* is *u*(*Panax ginseng*), whereas the marginal distribution function of AMK is *v*(AMK). Also, the tail of the density function in the graph has thicker morphological features, which better captured the tail correlation characteristics between *Panax ginseng* and AMK. This result also indicated that the model of *Panax ginseng* and AMK established by the *t*-copula function better reflected the correlation between *Panax ginseng* and AMK. Furthermore, Table 7 shows that the sum of the Kendall rank correlation coefficients of the *t*-copula function was 0.7403, whereas that of the Spearman correlation coefficient was 0.8958, indicating that *Panax ginseng* and AMK doses had robust nonlinear and positive correlations. Hence, when the doses of *Panax ginseng* and AMK changed greatly, the synergy between the two increased as well (Figure 7).

## 4. Discussion

Results showed that the composition of the *Panax ginseng* and AMK drug pair remained unchanged; however, different clinical manifestations were produced because of different dosages of the two drugs. Doses of *Panax ginseng* and AMK account for the difference in treating different indications. In treating diabetes mellitus and insomnia, measuring *Panax ginseng* and AMK showed a 1 : 1 relationship, whereas in treating diarrhea, the relationship was approximately 1 : 2. Moreover, *Panax ginseng*-AMK was used to treat different indications, and its medication was different [36]. For example, the effect of the Sijunzi decoction (*Panax ginseng*, AMK, *Poria*, and licorice) in treating diabetes mellitus was confirmed [8–10]. Among them, the dose ratio of *Panax ginseng* and AMK was approximately 1 : 1. Reports from *Jingyue Quanshu* stated that the Shenshu decoction (*Panax ginseng*, AMK, *Astragalus*, etc.) was used for diarrhea; however, it did not recommend the proportion of the drug to be used [8]. Thus, the combination of *Panax ginseng* and AMK (Guipi decoction: *Panax ginseng*, AMK, *Astragalus*, and *Angelica*) can also be used to treat insomnia with a significant effect and high safety, but the rule of the dose is around 1 : 1 [37].

The copula function was first proposed by Sklar [38]. It is a type of function that connects joint distribution functions with their respective marginal distribution functions, also called the connection function. The copula function can capture nonlinear, asymmetric, and tail correlations between variables [39] and has been used to study the dose relationship that exists between the clinical combinations of Western medicine. For example, Yin and Yuan proposed a Bayesian adaptive dose discovery design on the basis of the copula model to study the synergistic effect of doses when two or more drugs were used in combination [19]. Gasparini also proposed a new type of risk function on the basis of the copula function to evaluate combination therapy's clinical relevance [20]. Additionally, copula has been proven for use in comparing the effects of two diagnostic methods [21]. Therefore, we innovatively used the copula function to explore the correlation between Chuanxiong and Danggui. The copula function model analysis results showed that, in the three groups of prescriptions for treating irregular menstruation, sores, and stroke, the doses of Chuanxiong and Danggui were all positively correlated. These results also indicate that the copula function model was suitable for analyzing the dose correlation in TCM [26].

This study found that the *Panax ginseng*-AMK drug pair dose did not conform to the normal distribution depicted by the normality test, which showed a nonlinear and asymmetric trend. The copula function was used to obtain the respective joint distribution and probability density functions by exploring the frequency diagrams of different indications. The results indicated as well that, in the three groups of prescriptions for treating diabetes mellitus, insomnia, and diarrhea, the doses of *Panax ginseng* and AMK were all positively correlated. That is, as the dose of one medicine increases, the other medicine also tends to increase. Also, when the dose of *Panax ginseng* and AMK changes greatly, the synergy between the two increases as well.

The drug pair is the smallest unit of drug compatibility in TCM, and it is the communication bridge between a single medicine and compound compatibility. Thus, it is the dose of essential core factors of drug compatibility in TCM [40]. Hence, combining the qualitative and quantitative exploration of the relationship between drug pairs is essential for drug compatibility research. The amount changes according to the syndrome, and the prescription is derived from the method. TCM also believes that the prescription dose can affect the efficacy of the drug, thereby increasing the primary function of the prescription under certain conditions. This concept is similar to drug-drug interactions in modern medicine [41]. Thus, the core of TCM is the quantity of the drug. The “quantity” includes prescriptions, decoration pieces, and affective components. Furthermore, changes in the amount of medicine in the ancestral prescriptions affect the extent of prescription effectiveness. These changes even change the compatibility of the prescription, including the status of the leading prescription drugs, so that the main symptoms and functions of the whole prescription are changed.

Therefore, the copula function family provides new methods for pharmacometrics analysis. However, which function to choose as the optimal function must be selected on the basis of research questions and the squared Euclidean distance algorithm. Also, to describe the relationship between *Panax ginseng* and AMK doses, this study selected the best copula function from the Archimedes function family (Gumbel copula function, Clayton copula function, and Frank copula function) and the elliptical copula function (Gaussian copula function and *t*-copula function). Furthermore, various copula and empirical distribution functions are used to solve the squared Euclidean distance. Thus, the optimal copula function selected under this standard was the shortest distance between the corresponding empirical copula functions. This method is similar to a previous research method conducted on three indications of Danggui-Chuanxiong drug pairs [26], which chose the Gumbel copula function as the most preferred. Hence, we recommended selecting the best copula function according to the drug pair and data analysis when selecting the optimal copula function.

TCM has accumulated a large amount of literature studies in past years of development, which contain rich medical theories and clinical prescriptions with definite curative effects [17]. By analyzing and sorting out the doses used by doctors of the past dynasties to study drug pairs' dose rules, we provided a more detailed drug dose reference and basis for modern TCM clinics. Meanwhile, it also laid the foundation for research from “quantitative change” (dose change) to “qualitative change” (effect change). Also, introducing data mining technologies and mathematical statistics provides effective technical methods for processing massive amounts of information resources.

## 5. Limitations of the Study

Compared with other studies, this study is not free from limitations. The prescriptions we selected were renowned ones in ancient books, which are used more clinically; however, there were prescriptions prepared by doctors, nonclassical prescriptions, and special prescriptions that were not included. Our selection was in the limitation of the objective reality of data mining, aside from the fact that the prescriptions of contemporary doctors were not fully shared. Additionally, we only explored the correlation between doses from the perspective of mathematics and statistics. Therefore, further studies should be conducted to explore the correlation between doses in pharmacological experiments and clinical practice and optimize the dose.

## 6. Conclusion

This study conducted data mining on ancient prescriptions, constructed a copula function model, and studied the correlation between classic drug combinations, *Panax ginseng*-AMK, in treating different indications. A positive correlation was observed between the doses of *Panax ginseng* and AMK in treating diabetes mellitus, insomnia, and diarrhea. From the perspective of mathematical statistics, this study confirmed that, in TCM prescriptions, dose application of the medicine pair was relevant and provided a basis for guiding traditional prescriptions' clinical use. Moreover, this study also showed that the copula function model can be used for data analysis on the compatibility of TCM and that more data mining methodological studies on the basis of this concept are required. Also, this study only analyzed three main indications. However, some common clinical diseases exist for which *Panax ginseng*-AMK is used, such as fatigue and stroke, but they are used less than these three indications. They are also clinically applied. Therefore, we will conduct data mining and analysis on other indications in the near future. Additionally, this study was based on the dual copula function to identify clinical applications and dose relationships between the two drugs. Future studies can thus use three-dimensional or multivariate copula functions to analyze the dose relationships of TCMs for three or more drugs.

## Figures and Tables

**Table 1:** Examples of *Panax ginseng* and AMK databases.

Dynasty	Prescription	Prescription source	*Panax ginseng* dose		AMK dose	
			Before	After	Before	After
Tang	Da Dingxin Wan	Waitai Miyao	3 Liang	123.93 g	1 Liang	41.31 g
Tang	Bushen Fuling Wan	Waitai Miyao	2 Liang	82.62 g	2 Liang	82.62 g
Song	Baishiying Tang	Shengji Zonglu	1 Fen	0.4 g	1 Fen	0.4 g
Song	Yuanzhi San	Taipingshenghuifang	7.5 Qian	30 g	7 Qian	30 g
Yuan	Jiaweiqianshi Baizhu San	Danxixinfa	0.5 Qian	2 g	0.5 Qian	2 g
Yuan	Bazhen San	Ruizutangfang	1 Liang	40 g	1 Liang	40 g
Ming	Erzhi Wan	Fushoujingfang	1 Liang	36.9 g	3 Liang	110.7 g
Ming	Eryang Dan	Pujifang	1 Liang	36.9 g	1 Liang	36.9 g
Qing	Baozhen Wan	Jiyanliangfang	1 Liang and 3 Qian	47.97 g	1 Liang and 5 Qian	55.35 g
Qing	Huajing Dan	Bianzheng Lu	0.5 Liang	18.45 g	1 Liang	36.9 g

**Table 2:** Conversion principles for prescription units from each dynasty.

Dynasty	1 Jin (g)	1 Liang (g)	1 Qian (g)	1 Fen (g)
Tang	661	41.31	1.721	0.17
Song	663	40	4.0	0.4
Song	663	40	4.0	0.4
Yuan	663	40	4.0	0.4
Ming	590	36.9	3.69	0.37
Qing	590	36.9	3.69	0.37

**Table 3:** Descriptive statistics of variables.

Indications	Variables	Number of cases	*M* (IQR)	95% CI
Diabetes mellitus	*Panax ginseng* dose	110	20.0 (35.5)	(18.6, 28.2)
	AMK dose		20.0 (31.4)	(20.8, 29.9)

Insomnia	*Panax ginseng* dose	78	19.2 (28.7)	(19.6, 27.3)
	AMK dose		19.2 (27.9)	(19.2, 27.0)

Diarrhea	*Panax ginseng* dose	91	15.5 (31.3)	(17.5, 25.2)
	AMK dose		30.0 (29.4)	(21.0, 29.6)

**Table 4:** Normality test.

Indications		Kolmogorov–Smirnov			Shapiro–Wilk		
		Variable	df	*P* value	Variable	df	*P* value
Diabetes mellitus	*Panax ginseng*	0.163	110	<0.001	0.854	110	<0.001
	AMK	0.138	110	<0.001	0.909	110	<0.001

Insomnia	*Panax ginseng*	0.138	78	<0.001	0.918	78	<0.001
	AMK	0.154	78	<0.001	0.918	78	<0.001

Diarrhea	*Panax ginseng*	0.148	91	<0.001	0.895	91	<0.001
	AMK	0.149	91	<0.001	0.909	91	<0.001

**Table 5:** Parameter estimation of the copula function.

Copula function	Squared Euclidean distance	Parameter estimation	Kendall correlation coefficient	Spearman correlation coefficient
Clayton copula function	0.0353	*α* = 6.9819	0.7773	0.9297
Frank copula function	0.0158	*α* = 16.7833	0.7850	0.9421
Gumbel copula function	0.0221	*α* = 4.2334	0.7638	0.9211
Gaussian copula function	0.0821	*ρ* = 0.8646	0.6649	0.8538
*t*-copula function	0.0090	*ρ* = 0.9789, *v* = 6.0473	0.8689	0.9563

**Table 6:** The copula function parameter estimation.

Copula function	Squared Euclidean distance	Parameter estimation	Kendall correlation coefficient	Spearman correlation coefficient
Clayton copula function	0.0247	*α* = 6.5391	0.7958	0.9207
Frank copula function	0.0214	*α* = 16.8550	0.6989	0.9426
Gumbel copula function	0.0276	*α* = 4.3915	0.8211	0.9264
Gaussian copula function	0.0472	*ρ* = 0.8902	0.7723	0.8810
*t*-copula function	0.0108	*ρ* = 0.9608, *v* = 4.0089	0.7858	0.9276

**Table 7:** The copula function parameter estimation.

Copula function	Squared Euclidean distance	Parameter estimation	Kendall correlation coefficient	Spearman correlation coefficient
Clayton copula function	0.0241	*α* = 5.8181	0.7442	0.9064
Frank copula function	0.0153	*α* = 13.3355	0.7370	0.9133
Gumbel copula function	0.0376	*α* = 3.2661	0.6938	0.8711
Gaussian copula function	0.0400	*ρ* = 0.8706	0.6726	0.8602
*t*-copula function	0.0117	*ρ* = 0.9179, *v* = 2.6696	0.7403	0.8958

## Data Availability

All the data used to support the findings of this study are available within the article; if one needs database information, the corresponding authors can be contacted.
